# Clinical evaluation of balloon dilation Eustachian tuboplasty in the Eustachian tube dysfunction

**DOI:** 10.1007/s00405-012-2243-9

**Published:** 2012-11-08

**Authors:** Dariusz Jurkiewicz, Dominik Bień, Kornel Szczygielski, Ireneusz Kantor

**Affiliations:** Department of Otorhinolaryngology, Military Institute Medicine, Szaserów 128 street, 04141 Warsaw, Poland

**Keywords:** Eustachian tube, Balloon dilation

## Abstract

The development of minimally invasive procedures such as the balloon dilation Eustachian tuboplasty (BET) is an alternative to the grommet tympanum membrane. BET is applied in the cases where, after elimination of all factors influencing the ET and middle ear functioning, no sufficient improvement is observed. The aim of this study was to present the therapeutic benefits of the BET method in the treatment of ETD caused by disorders in the middle ear ventilation. The BET procedure was offered to four patients (3 men and 1 woman) after subjective, physical, otorhinolaryngological and audiometric examinations including pure tone audiometry, tympanometry and pressure-swallow test. As the method was novel, preinterventional CT angiography of the carotid arteries was performed in all patients. Any complications were noticed during and after the procedure (bleeding or damage of regional mucosa) in any patients. Our clinical studies assessed the feasibility and safety of the BET during a short-term period—only a 6-week observation. Although patients revealed a significant improvement of ET score, longer long-term studies are necessary to determine whether this method will demonstrate lasting benefits and safety in the treatment of chronic Eustachian tube dysfunction. In other investigations, improvement was found to be time dependent.

## Introduction

Hearing and its dysfunctions irrespective of their causes determine the quality of life. Even a slight hearing loss, feeling of blocking or check-a-block in the ear provide discomfort independent of the reasons for the disorders. They may be presbyacousis, chronic inflammation, proliferative process or posttrauma changes. One of the elements important in the process of hearing is the proper functioning of the Eustachian tube (ET). The Eustachian tube dysfunction (ETD) often co-exists with a subjective feeling of hearing loss. Although that we know anatomopathological causal of ETD, the diagnosis and treatment are persistent and laborious.

Result of ETD in infantile is the adenoids or/and pathological secretion within the rhinopharynx due to repeated infections of upper airways, regardless of age, the pathogenesis of ETD, chronic infections of the upper airways, allergy, gastroesophageal reflux, mechanical obstruction (e.g., rhinopharynx carcinoma), anatomical abnormality (e.g., palatoschisis, gothic palate, etc.), primary ciliary dyskinesia or different neuromuscular dysfunctions regardless of its courses [1, 2].

In most common pathological causes, pharmaceutical treatment with anti-histaminic or anti-inflammatory medicaments (including locally or generally used glucocorticosteroids) as well as a proper nasal toilet is often affective. If pharmacological treatment is ineffective while internal ear ventilation disorders caused by poor pathology of ET and chronic inflammatory process with exudation lead to partial hearing loss, surgery should be performed. There is a necessity to remove any pathological changes within the nose and the rhinopharynx and input the grommet tympanum membrane to remove the exudation from the ear. One of the methods is a less invasive procedure, the tuboplasty of the Eustachian tube (BET—balloon dilation Eustachian tuboplasty) introduced thanks to the development of endoscopic technology in laryngology. BET is applied in the cases where, after elimination of all factors influencing the ET and middle ear functioning, no sufficient improvement is observed.

## The objective

The aim of the study was to present the therapeutic benefits of the BET method in the treatment of ETD caused by disorders in the middle ear ventilation.

## Materials and methods

The BET procedure was approved by Ethics Committee of Military Institute of Medicine and was performed in accordance with the ethical standards laid down in the 1964 Declaration of Helsinki. All patients gave their informed consent prior to their inclusion in the study.

The BET procedure was offered to patients after subjective, physical and otorhinolaryngological examinations. The following clinically vital symptoms of poor ET function were recognized: feeling of lasting or periodic, but without regression to the norm, uni- or bilateral partial hearing loss as well as feeling of obstruction or clicking noises, which are reversible or not, after the Valsalva maneuver, yawning or swallowing.

Standard otorhinolaryngological of nose and rhinopharynx examination were followed by a transnasal endoscopic evaluation and microscopic examination of the ears. The Valsalva maneuver was carried out in all patients. Additionally, all patients underwent audiometric examinations (pure-tone audiometry, tympanometry and pressure-swallow test). Average air-bone gap for 500, 1,000 and 2,000 was evaluated. At the moment of qualification for the procedure, all patients denied any dysfunctions on their circulatory and respiratory systems, did not take any drugs at present, did not suffer allergy, any head or acoustic trauma or undergo any transnasal operations in the past.

**Fig. 1:**
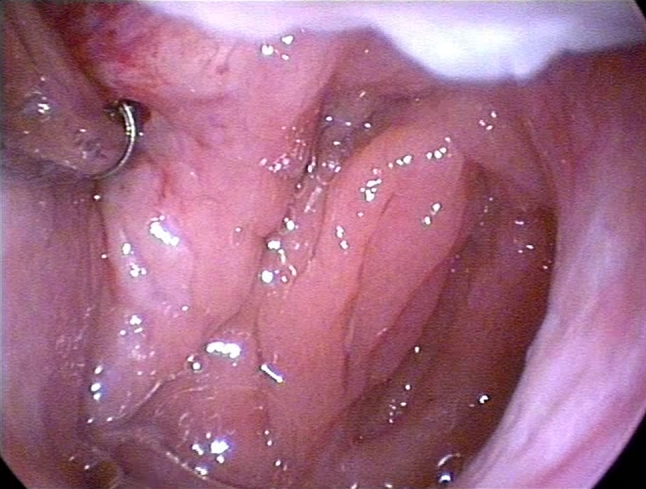
The balloon catheter was pushed 2 cm into the ET. After the balloon had been positioned correctly, the dilation was applied to a pressure of 10 bars for 2 min (using saline solution)

As the method was novel, preinterventional CT angiography of the carotid arteries was performed in all patients. All patients were treated with BET under general anesthesia. A specially designed 600 µm diameter balloon catheter (Spiggle and Theis Company) was applied in the procedure. The catheter was inserted into the pharyngeal orifice of ET, in the first instance (Fig. 1). The balloon catheter was pushed 2 cm into the ET. After the balloon had been positioned correctly, the dilation was applied to a pressure of 10 bars for 2 min (using saline solution). Then, the solution from the balloon was aspirated, and the catheter was removed. Seeing that there were no complications during and after the procedure (bleeding or damage of regional mucosa) in any patients, examination of mucous membrane in the place of dilation was unnecessary. Furthermore, endoscopic inspection was unrealizable.

## Results

BET was offered to four patients (3 men and 1 woman). Three of them had bilateral ET dysfunction. We did not notice any complications during and after the procedure (bleeding or damage of regional mucosa) in any patients.

Postoperative hearing examinations were performed at 1 week and 6 weeks after BET (Table 1). In the chart, there are results of objective method (tympanometry) and subjective methods (the air–bone gap, Valsalva maneuver and PST). Most of the patients have reported hearing improvement which resulted in reducing of the air–bone gap. One patient (C.M.) has not reported any hearing improvement of the left ear which meant no changes in the air–bone gap. Similar results in hearing improvement were observed in tympanometry. According to the chart, three patients showed improvement from type-B to type-A tympanogram including one person (C.M.) with no changes in the left ear tympanogram. In one case (P.W.), initial type-C tympanogram has showed improvement with the right ear and type-A tympanogram with the left ear. As a result of Valsalva maneuver conducted after 1 week and then after 6 weeks, three patients reported hearing improvement though the changes were reported at different time and to a different degree. PST test results have demonstrated similar findings. One person (P.W.) required no Valsalva maneuver to hear better.

**Table 1:** 

	Cochlear reserve		Tympanometry		Valsalva maneuver		PST	
	Right ear	Left ear	Right ear	Left ear	Right ear	Left ear	Right ear	Left ear
F.W.								
Before	20		B		+		−	
1 Week after	20		B		+		−	
6 Weeks after	5		A		+		+	
P.W.								
Before	25	20	C	C	−	−	−	−
1 Week after	20	20	C	C	−	−	−	−
6 Weeks after	10	10	AS	A	0	0	+	+
C.M.								
Before	10	15	B	C	−	−	−	−
1 Week after	10	15	C	C	−	−	−	−
6 Weeks after	5	15	A	C	+	−	+	−
D.Ł.								
Before	25	30	B	B	−	−	−	−
1 Week after	20	15	C	C	+	+	−	−
6 Weeks after	10	10	A	A	+	+	+	+

Valsalva maneuver

[−] Lack of hearing improvement

[+] Improvement of hearing

[0] Patient did not have to make the Valsalva maneuver = good hearing

PTS

[−] Blocking of the Eustachian tube

[+] Proper functioning of the Eustachian tube

While conducting BET procedure, there were no other methods available to objectify the results.

Patient F.W. (61 years old) assigned to right-hand BET. He reported temporal, ipsilateral, muco-serous exudate in the middle ear (without exudate for three years) and hearing loss which was reversible after the Valsalva maneuver. Periodical treatment against idiopathic rhinitis. Microscopic examination of the ears: left tympanic membrane anatomically and functionally without changes, right tympanic membrane-abnormal position (especially pars flaccida-retraction), single cicatrices and calcifications, residual reflex. Endoscopic examination of nose and rhinopharynx: small edema of mucous membrane. Otherwise no change. Valsalva maneuver on the right side-negative, on the left side-positive. One week after operation the patient reported reduced feeling of hearing loss. Microscopic examination of the ears: present state as before operation. Six weeks after operation without hearing loss. Valsalva maneuver was not obligatory. Microscopic examination of the ears: tympanic membrane in correct position. The patient reported reduced feeling of hearing loss.

Patient P.W. (61 years old) assigned to bilateral BET. Medical history: chronic, persistent mucoserous otitis despite pharmacological treatment, bilateral hearing loss. Bilateral grommet tympanic membrane three times (the last one was removed 4 years before BET), the feeling of blocked ears, ringing and increased pressure in both ears was reported. Status postcardiac infarct (2001), without pharmacological secondary prevention. Microscopic examination of the ears: retraction of tympanic membranes (especially pars flaccida), single cicatrices in the both ears. Endoscopic examination of nose and rhinopharynx: small edema of mucous. Otherwise no change. Valsalva maneuver in the both side—negative. One week after operation reduced feeling of hearing loss was reported. Microscopic examination of the ears: present state as before operation. Six weeks after operation without hearing loss. Valsalva maneuver was unnecessary. Microscopic examination of the ears: tympanic membranes in correct position.

Patient C.M. (38 years old) assigned to bilateral BET. Medical history: the feeling of blocked ears, increased pressure in both ears and primary hypertension—actually without drugs. Microscopic examination of the ears: tympanic membranes in abnormal position (retraction pars flaccida and pars tensa) in the both ears. Endoscopic examination of nose and rhinopharynx: small edema of mucous. Otherwise no change. Valsalva maneuver in the both side—negative. One week after operation reduced feeling of hearing loss was reported, especially in the right ear. Microscopic examination of the ears: present state as before operation in the left ear, smaller retraction in the right ear. Six weeks after operation felling of hearing loss in the left ear at the same level, in the right ear improvement was reported. Valsalva maneuver ought to be done. Microscopic examination of the ears: present state as before and one week after operation.

Patient D.Ł. (23 years old) assigned to bilateral BET. Medical history: chronic, persisting, mucoserous otitis despite pharmacological treatment, the feeling of blocked ears, bilateral hearing loss treated with catheterization of ET (several times, the last one two weeks before the operation without positive results), asthma and Scheuermann’s disease—without drugs since 2007. Valsalva maneuver in the both side—negative. Microscopic examination of the ears: retraction of tympanic membranes, level of liquid in middle ear in the both ears. Endoscopic examination of nose and rhinopharynx: small edema of mucous. Otherwise no change. One week after operation reduced feeling of hearing loss was reported. Microscopic examination of the ears: smaller retraction in the both ears. Six weeks after operation without hearing loss. Valsalva maneuver ought to be done occasionally. Microscopic examination of the ears: tympanic membrane in correct position. Reduced feeling of hearing loss was reported. Level of the postoperative pain in all patients just and in the first day after operation were between 0 to 2 points (in 10 steps scale of VAS where 0—without pain, 10—maximum of pain).

## Discussion

The development of minimally invasive interventions in the fields of urological, otolaryngological, gastrointestinal or vascular and cardiac procedures has become a fact [1, 2]. The widespread introduction of microcatheters, endoscopic tools with simultaneous improvements in data collection and transmission accompany “classical” operative techniques—Eustachian tuboplasty with stenting materials [3], laser Eustachian tuboplasty [4] or microdebrider Eustachian tuboplasty [5]. Transcanal, preauricular, transmastoid or middle fossa approach has not become a routine procedure [6, 7].

Already, in 1983, Yamashita utilized the fiberscope with an inflational canal to widen pharyngeal orifice and lumen of the ET [8]. In 2010, Ockerman and co-workers described the BET as a new method for treating patients with ETD [1, 2]. The method, which is technically easy to perform, significantly helped to improve ET functioning (eight patients with BET). To compare the pre- and postoperative results, they used the technique of tubomanometry (TMM)—a new method to assess ETD. TMM procedure allows to compare pressure equalization function of normal subjects and those with ETD objectively. A few other techniques like sonotubometry with perfect sequences (PSEQ) were described in order to estimate ETD accurately [9]. PSEQ seems promising but needs validation.

The BET procedure is difficult because of the variety of ETD mechanisms leading to its creation. Our clinical studies assessed the feasibility and safety of the BET in a small patient cohort during a short-term period—only a 6 weeks observation. It is in accordance with results of studies of balloon dilation techniques including small groups of patients described by other authors up to date. Although patients revealed a significant improvement of ET score, longer long-term studies are necessary to determine whether this method will demonstrate lasting benefits and safety in the treatment of chronic Eustachian tube dysfunction. In other investigations, improvement was found to be time dependent.
